# Stem cells and common biomaterials in dentistry: a review study

**DOI:** 10.1007/s10856-022-06676-1

**Published:** 2022-06-18

**Authors:** Seyed Ali Mosaddad, Boshra Rasoolzade, Reza Abdollahi Namanloo, Negar Azarpira, Hengameh Dortaj

**Affiliations:** 1 Student Research Committee, School of Dentistry, Shiraz University of Medical Sciences, Shiraz, Iran; 2 Student Research Committee, Department of Pediatric Dentistry, School of Dentistry, Shiraz University of Medical Sciences, Shiraz, Iran; 3 Dentistry Department, Bogomolets National Medical University, Kyiv, Ukraine; 4 Transplant Research Center, Shiraz University of Medical Sciences, Shiraz, Iran; 5 Department of Tissue Engineering, School of Advanced Medical Sciences and Technologies, Shiraz University of Medical Sciences, Shiraz, Iran

## Abstract

Stem cells exist as normal cells in embryonic and adult tissues. In recent years, scientists have spared efforts to determine the role of stem cells in treating many diseases. Stem cells can self-regenerate and transform into some somatic cells. They would also have a special position in the future in various clinical fields, drug discovery, and other scientific research. Accordingly, the detection of safe and low-cost methods to obtain such cells is one of the main objectives of research. Jaw, face, and mouth tissues are the rich sources of stem cells, which more accessible than other stem cells, so stem cell and tissue engineering treatments in dentistry have received much clinical attention in recent years. This review study examines three essential elements of tissue engineering in dentistry and clinical practice, including stem cells derived from the intra- and extra-oral sources, growth factors, and scaffolds.

## Introduction

Stem cells (SCs) are normal, undifferentiated cells that, if exposed to the proper signal, can multiply, produce, and differentiate into a variety of somatic cells in the laboratory and living organisms. Various SCs inside the body are involved in maturation and repair in adult organisms [1]. The unlimited potential of these cells to produce physiological cells has made them be replaced by recombinant or primer cells [2].

Oral tissues are a rich source of SCs, which have attracted dentists’ attention because of their easy access to other SCs. These cells have unique capabilities making them of great importance in tissue engineering [3, 4], regeneration, or the replacement of damaged or diseased tissues [4, 5]. In dentistry, there are problems such as alveolar bone resorption for patients following tooth extraction or loss due to periodontal disease, dental caries, and tooth fractures caused by trauma. Moreover, in individuals losing their teeth, it leads to bone loss, especially in the lower jaw, thereby making such individuals lose the treatment option of implant placement [6, 7]. Following such problems, stem cell tissue engineering therapies to repair large defects in periodontal tissue and alveolar bone to replace lost teeth seem to be of paramount importance [8–10].

Various studies on SC-based tissue engineering and the regeneration of oral and dental tissues and organs have been performed for clinical and dental applications in animal and laboratory models [11–13]. However, more invivo studies are recommended to reach further definite results [14, 15]. Given that basic research is required for treatment before evaluating SCs in clinical trials and also given the relatively new role of SCs in dentistry, obtaining ideal SCs, depending on the different locations of the mouth, jaw, and face, is not well described. Dental stem cells (DSCs) are attractive for stem cell transplant therapy approaches because of their simple separation, high flexibility, immunomodulatory properties, and multi-potential capabilities. The use of appropriate scaffolds filled with desirable biomolecules such as growth factors and cytokines can improve the proliferation, differentiation, migration, and functional capacity of DSCs.

Appropriate scaffolds full of desirable biomolecules such as growth factors and cytokines can improve the proliferation, differentiation, migration, and functional capacity of DSCs and optimize cell morphology to construct tissue structures for specific purposes. Since DSCs are a promising cellular source for tissue engineering, especially for repairing teeth, bones, and nerve tissues, the present study aimed to identify more DSCs and their therapeutic applications.

## Pluripotent stem cells (emryonic stem cells/ induced pluripotent stem cells)

Embryonic cells or induced cells are the main pluripotent stem cells (PSCs), which can produce themselves and a variety of somatic adult cells in vitro and in vivo [16]. Due to their unlimited renewal, these cells are used clinically in evolutionary biology, biological research, regenerative therapies, and pharmaceutical experiments in dentistry [17].

There are two types of SCs: [1]. Embryonic SCs (ESCs) taken from the cells of the inner layer of the embryo before implantation. They were first isolated from mice and then from other species such as rats, humans, and monkeys [13]. Human-derived ESCs are known as pluripotent, meaning that they can form different types of cells in the body [18], and [2]. Induced Pluripotent SCs (iPSCs) are formed by reprogramming adult somatic cells and converting them to SCs. The reprogramming technology on somatic cells was first performed in mice and then in human cells [19]. They are few and are often located deep in the tissue, making them somewhat difficult to identify, isolate, and grow in vitro [20].

Regarding the application of ESCs in dentistry, the controlled differentiation of PSCs to specific ratios of oral tissues and organs such as mucosa, alveolar bone, periodontal tissues, and teeth in vitro and in vivo is not unexpected. However, researchers in this field have faced two obstacles, including ethical issues and technical problems. Because ESCs are allogeneic, they may be immunologically incompatible between donors and recipients [21–24].

iPSCs are more accessible in terms of dental applications than ESCs because they can be extracted from tissues easily accessible to dentists. iPSCs cells originate from various oral mesenchymal cells, including SCs from apical papilla [25, 26], dental Pulp SCs and SCs from human exfoliated deciduous teeth [26, 27], tooth germ progenitor cells [28], buccal mucosa fibroblasts [29], gingival fibroblasts [30, 31], and periodontal ligament fibroblasts [32].

The role of iPSCs is highlighted in regenerating missing jawbones, periodontal tissue, salivary glands, and lost teeth [33]. In the mouse model, iPSCs and enamel matrix derivatives mainly enhance periodontal regeneration by promoting the formation of cementum, alveolar bone, and periodontal ligament [34]. In an in-vitro study, Duan et al. differentiated mouse iPSCs into ameloblasts and odontogenic mesenchymal cells, and this was a useful approach to dental bioengineering strategies [35].

Due to the limitations of the ESCs research, researchers tend to continue their research on adult SCs. They obtain mature SCs from many tissues such as cord blood, skin, bone marrow, hair follicles, striated muscles, tooth pulp, periodontal fibers, retina, and others [36, 37]. The second part of this review article examines adult stem cells and their application in dentistry.

## Adult stem cells

They are undifferentiated cells in the margins of the differentiated cells of body tissues and organs and can regenerate and differentiate into different types of specific cells, tissues, or organs. The primary roles of these cells in an organism are to support and repair the tissues from which they are derived [38].

### Bone marrow-derived MSCs (BMSCs)

The BM cell is a major source of adult SCs. BMSCs, which can differentiate into several cell lines, are acceptable candidates for repairing tooth and bone tissues [39]. BMSCs can be isolated from the iliac crest and the orofacial bones. BMSCs isolated from the iliac crest, known as the primary source of BMSCs, can be distinguished into myogenic, chondrogenic, osteogenic, adipogenic, and non-mesenchymal neurogenic lineages [11, 40].

Since it is not possible for physicians to separate BMSCs from the bone marrow of the iliac crest of donors without surgery, and given that it is comfortable in terms of separation, it is an invasive separation method. This can be considered as one of its drawbacks [41, 42]. However, this method has been used for many years in tooth bone reconstruction despite such drawbacks. Another drawback is the relationship between donors’ age and the bone potential of BMSCs isolated from the iliac crest [43]. Age is a critical factor for bone tissue engineering and the clinical effectiveness of bone formation, so it is recommended that the donor’s age be considered. This is because cells naturally tend to age and lose their multiple differentiation potential over time [44].

Such problems have made researchers conduct more extensive studies to isolate human BMSCs. Human BMSCs can be achieved through bone marrow spinal cord aspirate (maxilla and mandible), which is possible during dental treatments such as dental implants, wisdom tooth extraction, cyst removal, and orthodontic osteotomy [45].

The advantage of oral bone BMSCs compared to iliac crown BMSCs is that there is no age limit for donors so that oral bone BMSCs can be received from patients aged 6–60 years, and age would not have much effect on the BMSC gene expression pattern [46].

Another noteworthy point is the differences in embryonic origin causing functional differences between oral and human iliac BMSCs [47, 48]. The embryonic origin of the maxillary and mandibular bones is the cranial nerve crest cells, and the embryonic origin of the iliac crown bone is the mesoderm. In terms of phenotype and function, oral BMSCs are different from the iliac coronary BMSCs. It has been documented that grafted bone obtained from the craniofacial area (membranous bone) has beneficial in autologous bone grafting in the skull and face and can also significantly increase the volume of endochondral bone (iliac crest) [49].

It has also been reported that the adipogenic potential of oral BMSCs is lower than that of iliac BMSCs [50]. This advantage of this factor is reducing the formation of unfavorable adipose during bone tissue regeneration. All of reviewed studies confirm the usefulness of oral BMSCs compared to iliac BMSCs for bone regeneration. However, due to the possibility of collecting a larger volume of iliac BMSCs than oral BMSCs, the use of BMSCs of iliac crown origin is more prevalent among professionals [51, 52].

### Dental tissue-derived stem cells

Oral epithelial stem cells (OESCs) and Mesenchymal stem cells (MSCs) are two types of mature SCs in tooth tissue. Dental tissues, including dental pulp and periodontal tissues, can regenerate and form restorative dentin due to their suitable conditions after dental operations. From these tissues, MSCs or SCs can be extracted [53].

**Fig. 1:**
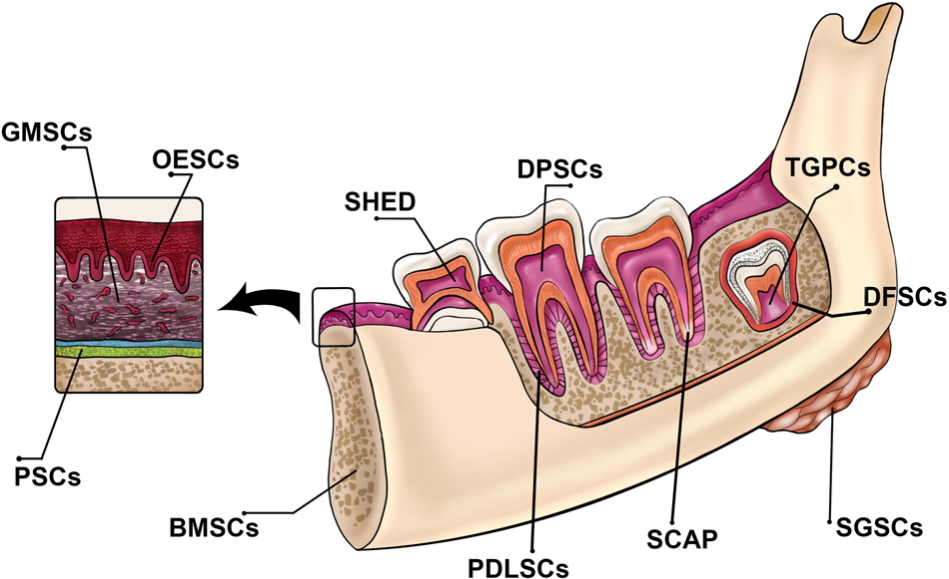
Adult stem cell sources in the maxillofacial and oral area. DFSCs: dental follicle stem cells; SCAP: stem cells of the apical papilla; OESCs: oral epithelial progenitor/stem cells; SHED: stem cells from human exfoliated deciduous teeth; BMSCs: bone marrow-derived MSCs from orofacial bone; DPSCs: dental pulp stem cells; PDLSCs: periodontal ligament stem cells; SGSCs: salivary gland-derived stem cells. TGPCs: tooth germ progenitor cells; PSCs: periosteum-derived stem cells; GMSCs: gingiva-derived MSCs

To date, various sources of MSCs have been identified in dental tissues, and SCs isolated from such sources have also been addressed [54]. The SCs of dental origin are as follows: Dental Pulp SCs (DPSCs), Dental Follicle Progenitor Cells (DFPCs), SCs from Exfoliated Deciduous Teeth (SHED), SCs from Apical Papilla (SCAP), Tooth Germ SCs (TGSCs), Periodontal Ligament SCs (PDLSCs), Tooth germ progenitor cells (TGPCs), and gingival mesenchymal stem/progenitor cells (GMSCs). Figure 1 summarizes all possible dental tissues as a source for stem cells.

#### Dental pulp stem cells (DPSCs)

DPSCs are SCs, which can be isolated from the pulp tissue of human extracted third molar by enzymatic digestion and can provide a typical fibroblast-like morphology [55]. In dentistry, they play a role in repairing and restoring teeth and have a high differentiation capacity.

DPSCs can differentiate into osteoblast, adipocyte, chondrocyte, muscle cells, melanoma cells, hepatocytes, and endothelial cells [56–58]. They can also differentiate into islet cell aggregates such as pancreatic islet and are an acceptable candidate for future diabetes treatments [59]. DPSCs can secrete anti-apoptotic and proangiogenic agents, which can be useful in the treatment of myocardial infarction [58]. When transplanted into SCID mice, these cells can form a pulp/dentin-like complex [60]. DPSCs can differentiate into active and functional neurons and have potentials for cell therapy in neurological diseases [61].

Markers expressed by DPSCs include CD9, CD10, CD13, CD29, CD44, CD49d, CD59, CD73, CD90, CD105, CD106, CD146, CD166, and CD271. Moreover, embryonic SCs markers are STRO-1, Nestin, Oct-4, Nanog, TRA-1-66, TRA-1-81, SSEA-3, and SSEA-4 [62, 63]. From an immunological viewpoint, Human leukocyte antigen G (HLA-G), hepatocyte growth factor (HGF), transforming growth factor-beta (TGF-β), interleukin-6 (IL-6), and prostaglandin E2 (PGE2), which are anti-inflammatory cytokines, are released from DPSCs [64, 65].

The production of indole amine 2, 3-dioxygenase (IDO), and nitric oxide (NO), which are essential for inducing maternal immunological tolerance against the fetus, also occurs by DPSCs. Accordingly, DPSCs also encompass immune regulatory properties [64]. DPSCs show great potentials for producing large volumes of the mineralized matrix, which opens a window of hope for being used in regenerative dental treatments. They can treat dental problems such as the scaly deciduous teeth of children, apical papilla, periodontal ligament fibers, and tooth follicle tissue [66, 67].

#### Dental follicle progenitor cells (DFPCs)

The tooth follicle is a loose mesenchymal tissue around the growing bud of the tooth and is involved in the formation of periodontal progenitor cells [68]. DFPCs isolated from the follicles of the human third molar present a fibroblast-like morphology. DFPCs can bone differentiate and are also adipocyte, chondrocyte, neural cells, periodontal, ligament, fibroblast, and hepatocyte-like cells (HLCs) [69].

They express the markers of mesenchymal stem cells, including Notch1, STRO-1, Nestin, CD105, CD90, CD73, CD59, CD44, CD29, CD13, and CD10 [70, 71]. These cells have immunosuppressive properties and also suppress the secretion of TGF-β and IL-6 [72]. They are also acceptable candidates for treating chronic inflammatory diseases [73].

STRO-1-positive dental follicle stem cells can differentiate into cementum in vivo. DFPCs also have the potential to differentiate and express cementoblast markers stimulated by bone morphogenic protein 2 (BMP-2), bone morphogenic protein 7 (BMP-7), and EMD in vitro [74, 75].

#### Stem cells from human exfoliated deciduous teeth (SHED)

SHEDs are mesenchymal cells inside the pulp tissue of scaly deciduous teeth [76]. SHEDs are highly proliferative and clonogenic in nature and generate sphere-like clusters [77]. These cells can differentiate into myocytes, chondrocytes, adipocytes, osteoblasts and odontoblasts, and nerve-like cells and have high plasticity. Morphologically like DPSCs and DFPSCs, they are similar to fibroblasts [78].

SHEDs lead to the expression of MSCs markers, including CD13, CD29, CD44, CD73, CD90, CD105, CD146, CD150, CD166, Oct4, Nanog, Nestin, SSEA-3, SSEA-4, and STRO-1 [79]. SHEDs differ from DPSCs in some aspects. Their difference is in their high proliferative capacity, bone formation and odontogenic ability in vivo, and inability to form pulp/dentin complex [80]. When transplanted into immunocompromised mice, these cells formed dentin-like tissues and reacted to dentin-specific sialo phosphoprotein antibodies [81]. Moreover, unlike DPSCs, SHEDs cannot differentiate into osteoblasts or osteocytes; however, they can induce host cells to differentiate into bone. By absorbing host cells, they induce the formation of a bone-like matrix with a layered structure [82, 83].

The transplantation of SHEDs to the striatum in mice with Parkinson’s has documented to improve disease-induced rotational movements partially, suggesting that SHEDs can be used as a source of postnatal stem cells in the treatment of Parkinson’s [84].

#### Stem cells from apical papilla (SCAP)

The apical papilla is a tissue gently attaching to the top of a growing tooth [85]. MSCs are present within the apical papilla of immature permanent teeth. During tooth growth, dental papillae become tooth pulp and help root growth. The apical papilla contains fewer cellular and vascular elements than the dental pulp; however, the cells in the apical papilla are more proliferative than those in the dental pulp [86].

SCAPs have a high proliferation rate and can be isolated from the human third molar. They can undergo osteogenic, adipogenic, chondrogenic, and neurogenic differentiation. It has been observed that after SCAP grafting to immunocompromised mice, a typical dentin-pulp-like structure begins to form due to the presence of odontoblast-like cells [85, 87].

Primary mesenchymal surface markers such as CD24, CD44, CD49d, CD51/61, CD56, CD73, CD90, CD105, CD106, CD146, STRO-1, Scleraxis, Nestin, and Survivin are expressed in SCAP, among which CD24 is a specific SCAP marker [70, 88]. SCAP also plays a role in suppressing the immune system by preventing T cell proliferation [89].

#### Tooth germ progenitor cells (TGPCs)

Tooth germ is the accumulation of progenitor cells making teeth and their tissues form [73]. Because the tooth germ of the third molar is formed after the age of 6 years, and the tissues remain from the embryonic period until then they remain undifferentiated. Hence, the proliferative capacity of these cells is extremely high [90]. TGPCs are relatively new SCs isolated from the human third molar. This cell can differentiate into chondrocytes, adipose, osteoblasts, odontoblasts, and neurons. In vitro, they show the ability to differentiate into liver cells, providing the grounds to cure liver diseases using these cells [70, 91].

Cells derived from the tooth germ of the third molar reflect the characteristics of MSCs. Human tooth germ cells can express surface antigens specific for MSCs such as CD166, CD105, CD106, CD90, CD73, CD44, CD29, and STRO-1, Nanog, Nestin, Oct-4, Sox-2, C-myc, and Klf4 [92]. TGPC is also involved in the expression of Nanog, Oct4, Sox2, klf4, and C-myc genes [93] and expresses markers associated with MSCs, including STRO-1, HLA class1, CD29, CD44, CD73, CD90, CD105, CD106, and CD166 [73, 94]. The hydroxyapatite/TGPC implants have indicated the formation of new bone in the presence of osteocytes in the newly formed bone matrix and the active cube-shaped osteoblast coating on the surface of the matrix [95].

TGSCs can differentiate into osteoblast, odontoblast, adipocyte, and neural cells due to their multipotency. The cartilage and bone differentiation of TGSCs is enhanced by F68, a pluronic block copolymer [70, 96]. Furthermore, the capacity of odontogenic differentiation can be increased by BMP-7 and osteogenic via BMP-2, which can be transferred to TGSCs by electroporation [97, 98]. Human-derived TGSCs have immune-regulating properties. Guzmán et al. showed the use of human-derived TGSCs as an immunosuppressive agent in mice [99].

#### Periodontal ligament stem cells (PDLSCs)

PDLSCs are SCs located in the area around the periodontal arteries surrounding the tooth. They are in charge of regenerating periodontal elements such as the alveolar bone, cementum, and ligament periodontal fibers. PDLSCs is a crestoriginated neural tissue [100].

These cells make the connection between bone and cementum. If grafted to the appropriate host, they can also form the PDL/cement structures [101]. PDLSCs provide the biological balance of teeth and the repair of damaged tissue [102]. These cells are available by enzymatic digestion from the periodontal ligament area. The cells obtained from this region have the characteristics of mesenchymal stem cells [103].

PDLSCs are morphologically and proliferatively similar to MSCs and are capable of expressing STRO-1, Scleraxis, CD166, CD146, CD106, CD105, CD90, CD73,CD59, CD49d, CD44, CD29, CD13, CD10, and CD9 markers [104]. PDLSCs can differentiate into bone, cartilage, adipose, and neuronal cells in vitro, and cementoblasts in vitro and in vivo [75, 105]. PDLSCs can suppress the immune system and can reduce the induction of Treg. They can also release IDO, HGF, and TGF-β [64, 89].

#### Oral mucosa-derived stem cells (OMSCs)

The oral mucosa includes stratified squamous epithelium above the connective tissue termed lamina propria. It is an area with vascularized tissue and the submucosa with adipose tissue, minor salivary glands, lymphatic tissues, and neurovascular bundles, which depend on the site [106]. The oral mucosa encompasses various forms of human adult stem cells, including the oral epithelial stem cells/progenitor, as the subpopulation of small oral keratinocytes (< 40 μm) [107]. Such cells are unipotently stem cells, suggesting that they can only develop into epithelial cells; however, they have clonogenicity and can reproduce a well-organized and highly stratified oral mucosal graft ex vivo [108, 109], indicating their effectiveness for intraoral grafting [110].

Other OMSCs are in the gingiva lamina propria directly connected to the underlying bone periosteum without intervening submucosa [111]. There are frequent resections in gingiva overlying the alveolar ridges and retromolar areas throughout general dental treatments, mostly achieved as a discarded specimen. Zhang et al. [32] could first characterize human gingiva-derived MSCs (GMSCs) with self-renewal, clonogenicity, and a multipotent differentiation ability comparable to BMSCs. GMSCs proliferate more quickly than BMSCs and exhibit a fixed morphology preserving their MSC features with long passaging [112]. According to Marynka-Kalmani et al. [113], OMSCs can be reproducibly produced from the human gingiva adult lamina propria and can differentiate into the lineages of the three germ layers in vitro. Accordingly, the gingival cells’ stemness can indicate the increased reprogramming effectiveness of gingiva-derived fibroblastic cells during the generation of iPSCs [114]. Furthermore, GMSCs/OMSCs offer more advantages in clinical settings due to their multipotency, clinical abundance, ease of isolation, and quick ex-vivo expansion.

#### Gingival mesenchymal stem/progenitor cells (GMSCs)

GMSCs can be easily removed from the gums with minimal pain and discomfort [115]. GMSCs include clonogenicity, self-renewability, multipotent differentiation capacity, and SC-like and immune-regulating features [116, 117].

GMSCs are involved in the expression of Oct-4, Sox-2, Nanog, Nestin, SSEA-4, HLA-ABC, Tra2-49, Tra2-49 and STRO -1 genes, and CD29, CD44, CD73, CD90, CD105, CD106, CD146, and CD166 [117]. GMSCs can have self-renewal, form connective tissue structures in vivo, and differentiate minerals, fats, and cartilage in vitro [3]. Wang et al. found that GMSCs acquire the ability to differentiate into osteogenesis in vivo after going through the incubation steps in vitro. Their findings promise the clinical use of GMSCs in tissue regeneration and repair [118]. Table 1 demonstrates various characteristics of dental tissue-derived stem cells.

**Table 1:** Dental stem cell characteristics

Stem cells	Positive markers	Negative markers	Other market	Differentiation potential	Reference
Dental Pulp Stem Cells	CD9, CD10, CD13, CD29, CD44, CD49d, CD59, CD73, CD90, CD105, CD106, CD146, CD166	CD14, CD31, CD34, CD45, CD117, CD133	STRO-1, Nestin, Oct-4, Nanog, TRA-1-66, TRA-1-81, SSEA-3/4	Osteoblast, odontoblast, adipocyte, chondrocyte, neural cells, muscle cells, melanoma cells, hepatocytes, endothelial cells	[57, 112, 113, 118, 262, 263]
Dental Follicle Progenitor Cells	CD10, CD13, CD29, CD44, CD59, CD73, CD90, CD105	CD34, CD45	Notch1, STRO-1, Nestin	Osteoblast, adipocyte, chondrocyte, neural cells, cementoblast, periodontal ligament, fibroblast, hepatocyte- like cells (HLCs)	[101, 262, 264, 265]
Stem cells from Human Exfoliated Deciduous teeth	CD13, CD29, CD44, CD73, CD90, CD105, CD146, CD150, CD166	CD14, CD19, CD34, CD43, CD45	STRO-1, Nestin, Oct-4, Nanog, SSEA-3/4	Osteoblast, odontoblast, neural cells, adipocyte, hepatocytes, endothelial cells	[266–268]
Stem Cells from Apical Papilla	CD24, CD44, CD49d, CD51/61, CD56, CD73, CD90, CD105, CD106, CD146, CD166	CD14, CD18, CD34, CD45, CD117, CD150	STRO-1, Scleraxis, Nestin, Survivin	osteoblast, odontoblast, neural cells, adipocyte	[269–271]
Tooth Germ Stem Cells	CD29, CD44, CD73, CD90, CD105, CD106, CD166	CD31, CD34	STRO-1, Nanog, Oct-4, Sox-2, C-myc, Klf-4, Nestin	Osteoblast, odontoblast, neural cells, adipocyte	[91, 272, 273]
Periodontal Ligament Stem Cells	CD9, CD10, CD13, CD29, CD44, CD49d, CD59, CD73, CD90, CD105, CD106, CD146, CD166	CD31, CD34, CD45	STRO-1, Scleraxis	Osteoblast, chondrocyte, adipocyte, neural cells	[101, 262, 264, 265]
Tooth germ progenitor cells	CD29, CD44, CD73, CD90, CD105, CD106, CD166	CD14, CD34, CD45	STRO-1, Oct-4, Nanog, HLA-1	Osteoblast, odontoblast, adipocyte, chondrocyte, neural cells, hepatocytes	[93, 94, 274, 275]
Gingival mesenchymal stem/progenitor cells (Oral mucosa-derived stem cells)	CD29, CD44, CD73, CD90, CD105, CD106, CD146, CD166	CD117, CD34, CD45	STRO-1, Oct-4, Nanog, Nestin, Sox-2, SSEA-4, HLA-ABC, Tra2-49/54	Osteoblast, adipocyte, chondrocyte, neural cells	[68, 115–117]

### Periosteum-derived stem/progenitor cells

The periosteum is a thick membrane of numerous cell layers covering almost the entire surface of each bone. The only parts not covered by this membrane are areas covered by cartilage. In addition to covering the bone and supplying blood, the periosteum also produces bone if properly stimulated [119]. The periosteum consists of two layers, an outer fibrosis layer consisting of elastic fibers and fibroblasts and an inner layer consisting of MSCs, fibroblasts and osteoblasts, and sympathetic nerves, which can differentiate into fat, osteoblasts, and chondrocytes. Moreover, the periosteum has ossifying properties due to the presence of bone progenitor cells, and if it stays healthy, it can produce new bone along small fibers and blood vessels [120]. Since the findings of previous studies have revealed that single-celled clone populations derived from adult human periosteum can differentiate into adipocytes, chondrocytes, osteoblasts, and skeletal myocyte lineage in vivo and in vitro, it is possible to ensure that derived cells from the periosseous tissues can be used in bone reconstruction and tissue engineering [121, 122].

One study found that cells derived from periosseous tissues to strengthen the sinus or alveolar ridge during implant placement yielded promising results in improving bone regeneration and lamellar bone production during the shortest time. Such findings could encourage dentists to use the periosteum to regenerate oral bones [123]. Accordingly, in the presence of large bone defects, the periosteum can be used as a source of precursor stem cells to regenerate bone.

### Salivary gland-derived stem cells

These glands are located around the mouth and throat, originate from the endoderm, and consist of acinar and duct epithelial cells with exocrine function [124]. The salivary glands are classified into two groups, major and minor. Major glands include the submandibular and sublingual parotid glands. Minor glands are mainly located on the roof of the mouth and lips; however, they are found in different areas of the throat and larynx [3]. In head and neck cancer, patients undergoing radiation therapy, unfortunately, experience irreversible dysfunction of the salivary glands, making the mouth dry and affecting individuals’ quality of life. This implies that stem cells in salivary glands are useful in the treatment of autologous transplantation in tissue engineering and direct cell therapy [11].

To date, in vitro studies have been conducted to isolate SCs in the salivary glands, and successful results have been achieved. For example, in one study, the researchers were to isolate salivary gland progenitor SCs from the submandibular glands of mice. They observed that the cells can express acinar, ductal, and myoepithelial cell lineage markers [125].

In another study, a specific population of SCs was isolated from the submandibular glands of mice using a laboratory floating sphere culture method. The findings of the study revealed that these cells can differentiate into the salivary gland and acinar cells producing mucin and amylase in vitro. Such findings also promising regarding the use of salivary gland stem cells to treat cancer patients in the head and neck area undergoing radiotherapy [126–128]. However, the primary culture of scattered cells involves several cells of different origins, including stromal cells, blood vessel cells, and parenchymal cells, which can make the selection of salivary SCs difficult. Accordingly, selecting cells with specific markers or those labeled with induced reporter proteins is necessary to isolate the main population of true SCs from the salivary glands [129].

### Adipose tissue-derived stem cells (ASCs)

ASCs are mature SCs derived from adipose tissue and can differentiate into mesenchymal and non-mesenchymal classes [129–131]. Adipose tissue has been of interest due to containing large amounts of SCs compared to bone marrow and their ability to bind to native and non-native hosts [132]. A noteworthy point is the easy access to large volumes of fat during the liposuction process [133]. ASCs and bone marrow stromal cells are similar in terms of gene expression and differentiation; however, ASCs have a higher potential to self-replicate [134].

Previous studies have indicated that SCs isolated from the adipose tissue of the rat abdominal cavity mimic the differentiation process of human adipose-derived SCs and can show the appearance of adipose, cartilage, bone, and nerve cells [135, 136]. Recent studies have documented that using this tissue as a source of MSCs has provided enormous potentials for tissue engineering applications and the production of natural scaffolding [137, 138].

ASCs show stronger osteogenesis than BMSCs as such they are expected to be an alternative source of MSCs for bone regeneration in dentistry [139–141]. Hence, ASCs can be used in the regeneration of oral facial bones, guided bone regeneration, and implant placement [142–144].

Regarding the effect of ASCs on dental pulp, ASCs transplantation leads to pulp regeneration in the root canal after pulpectomy in dogs [145]. Another study suggested that the transplantation of autologous ASCs with an inorganic bovine bone scaffold (bio-Oss1) results in new ossification. It also enhanced implant ossification following the vertical increase in the calvarial bone of rabbits, suggesting that ASCs can strengthen vertical alveolar bone in implant treatment [146].

**Fig. 2:**
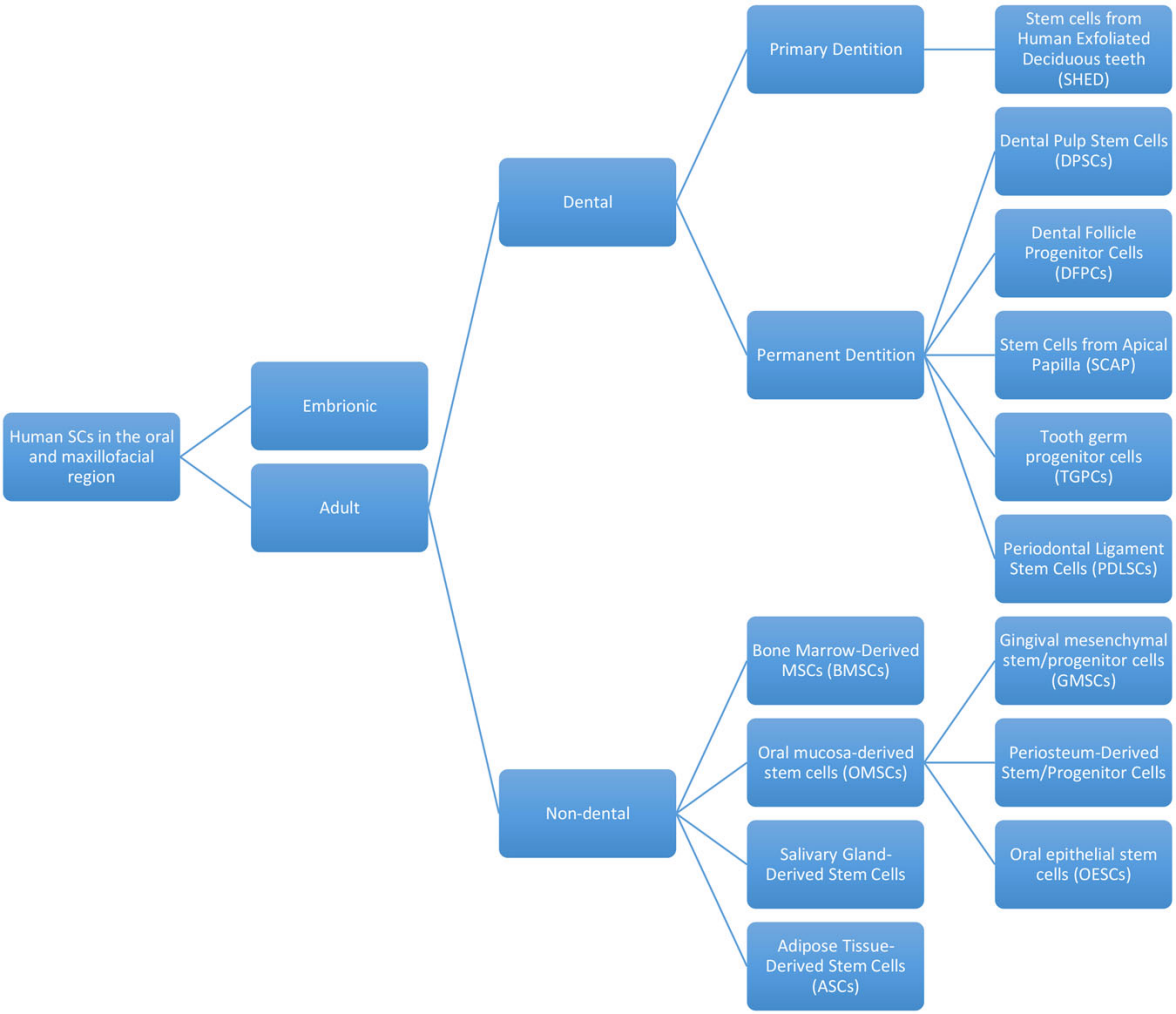
Human stem cells classification in oral and maxillofacial region

Regarding the role of ASCs in periodontal tissue regeneration in a laboratory study, ASCs obtained from a mouse model cultured in an environment with dental follicle cells containing non-collagenous proteins revealed cement blast features [48, 147]. Figure 2 summarizes the previously-mentioned stem cells classification.

## Biomaterials and scaffolds used with DSCs

Cells in a three-dimensional microenvironment demonstrate the ability of cell-cell and cell-matrix interactions to maintain normal cellular behavior. Since cell culture plates restrict cells to a 2D level and prevent their necessary free interactions, and on the other hand, because the developed cells were isolated from the culture plate, they lose a large number of cell-matrix and cell/matrix interactions, leading to lower survival rates and poor in vivo transplantation [148]. Accordingly, it is necessary to develop 3D systems and create a more natural environment for cell growth are [149, 150].

To succeed in tissue engineering, to repair, regenerate, and improve the function of defective tissues, the proper selection of scaffolding materials, stem cell types, and bioactive factors is of paramount importance. A proper scaffold increases the ability of DSCs to repair and regenerate damaged organs by improving the proliferation, differentiation, adhesion, and migration of DSCs [151]. Biomaterials are one of the essential components in making scaffolds. By combining scaffolds with different stem cells, tissue bioengineering offers promising results in regenerating damaged tissues [152].

The characteristics of an ideal biomaterial for bonding with stem cells are its biocompatibility in the first stage and then their ability to exchange gas and nutrients, their ability to protect cells against immune system invasion and external stress [153], pore size [154], stability [155], electrical conductivity [156], porosity [154], connectivity, and the ability to create proper crosstalk between stem cells and adjacent cells [157]. Since DSCs are an acceptable source of cells for regenerating teeth, bones, and nerve tissue, combining DSCs with a suitable scaffold for cell transplantation can provide remarkable results. Two main approaches to this combination are cell-based tissue engineering and the cell-free approach [158].

When a bioactive scaffold with growth and differentiation factors is implanted in relevant tissues, it can induce resident stem cells and their promotion, reproduction, and differentiation [159]. Various morphogens/growth factors, as environmental cues, significantly affect the behavior of DSCs implanted in scaffolds and play a key role in the success of regenerative therapies [160, 161]. If different biomaterials are pretreated with proteins such as BMP, sialoprotein, fibronectin, and osteopontin, it improves the DSCs’ behaviors. They promote the function of DSCs by increasing their adhesion, differentiation, proliferation, and migration and ultimately improve the formation of new tissues [162, 163].

Some sources to produce scaffolds for the teeth repair and reconstruction are natural biomaterials, including collagen [164], gelatin [154], fibrin, and silk [165] with protein structure and alginate [166], hyaluronic acid [167] with polysaccharide structure, and synthetic biomaterials, including polyglycolate/poly-l-lactate [168], polycaprolactone-poly glycolic acid [169], polylactic acid-co-polyglycolic acid [170], polycaprolactone /gelatin/nano-hydroxyapatite [171], nano-hydroxyapatite/collagen/poly-l-lactide [172], and polyethyl methacrylate-co-hydroxyethyl acrylate [173].

### Natural biomaterials

Collagen is a major component of the extracellular matrix (ECM), expressed in bones, teeth, and the brain. This biomaterial has poor mechanical, chemical, and thermal stability and has a high decomposition rate. However, when presented as a collagen scaffold, it provides high biocompatibility and controllable biodegradability for bone tissue engineering [174, 175].

As the second and the most abundant natural semi-crystalline polysaccharide, Chitosan is one of the most widely used biomaterials in tissue engineering, including periodontal tissue regeneration [176, 177]. Chitosan/gelatin scaffolds are associated with a significant increase in the survival and differentiation of DSCs; hence, this type of scaffold can increase the formation of hydroxyapatite-rich nanocrystalline calcium phosphate in immunocompromised mice [178]. Chitosan has revealed less support for the growth and differentiation of human DSCs compared to collagen and gelatin [179]. Fibrin is known as a non-toxic biomaterial scaffold connecting various biological surfaces to regenerate bone and nerve tissue [180]. Due to its low mechanical stiffness, fibrin scaffolds have limitations on directly implanting cells into damaged tissue [181, 182]. A summary of the natural scaffolds in oral tissue engineering is presented in Table 2.

**Table 2:** Natural polymers used as scaffold in dental tissue engineering

No.	Author and Year	Defect location	Type of stem cell	Type of scaffold	Growth factor	Outcome
1	Zou et al. [276]	Calvaria	BMSCs	Gelatin Sponge	HIF-1α	After 8 weeks, histological examination showed bone and vascular formation
2	Joon et al. [277]	Mandible	MDSCs	Collagen sponge	BMP 2	After 2 weeks, 95 to 100% of the lesions were repaired
3	Miranda et al. [278]	Tooth sockets	BMSCs	Chitosan-gelatin	–	After 21 days alveolar bone and epithelial healing were established
4	Kato et al. [279]	Periodontal wound healing	–	Collagen Hydrogel	BMP-2	BMP and collagen hydrogel scaffold implantation facilitated the reestablishment of periodontal attachment
5	Al-Salihi [280]	Mandible	BMSCs	Coral	–	After 3 months the histology showed mature bone formation
6	Weng et al. [281]	Subcutaneous implantations	BMSCs	Coral	–	New bone formation and vascularization were observed after 12 weeks
7	Dudas et al. [282]	Calvaria	ADSCs	Gelatin foam	BMP2	After 6 weeks of repair, 65% of the lesion was confirmed by radiography
8	Ito et al. [283]	Mandible	BMSCs	PRP	–	Mature bone formation was seen after 2 weeks
9	Smiler et al. [284]	Maxillary sinus	–	Algae & βTCP	–	In the use of Algea polymer, more bone than βTCP was formed after 4 months
10	Cui et al. [285]	Parietal	ADSCs	Coral	–	After 24 weeks, the radiograph showed repair of most of the lesion
11	Kim et al. [286]	Calvaria	BMSCs	Hyaluronic acid	BMP2	After four weeks, mature bone formation in histology Vascular factors were observed
12	Usas et al. [287]	Calvaria	MDSCs	Collagen & Fibrin gel	BMP4	After 6 weeks, collagen showed more bone repair
13	Park et al. [288]	In vitro	hDPCs	Glycol chitin–based thermoresponsive hydrogel	Enamel matrix derivative	GC-TRS allowed the proliferation and odontogenic differentiation of hDPCs useful in dentin regeneration
14	Zhang et al. [289]	Calvaria	BMSCs	Silk fibroin	BMP7	After 4 weeks new bone in the margins and islets in The center of the lesion showed
15	Lucaciu et al. [290]	Parietal	BMSCs	Deer Horn	–	After two or four months, histology examination showed bone formation
16	Yamada et al. [291]	Maxilla	BMSCs	PRP	–	After 3 months, the bone height had increase
17	Kohgo et al. [292]	Mandible	BMSCs	PuraMatrix	PRP	After 8 weeks bone has formed around the implant
18	Ye et al. [293]	Calvaria	iPSCs	Silver	SATB2	New bone formation was observed after 5 weeks
19	Tong et al. [294]	Mandible	–	Silk fibroin-chitosan	TGF-β1	After 8 weeks biocompatibility and extensive osteoconductivity and osteogenesis were observed
20	Florczyk [295]	Calvaria	MSCs	Chitosan–alginate	BMP-2	The applied scaffold demonstrated the greatest osteogenic properties

### Synthetic biomaterials

In contrast, bioceramic scaffolds have high mechanical stiffness, and due to their chemical and structural similarity to native bone, they have high biocompatibility and excellent bone conductivity. Porous and spongy scaffolds can deliver more DSCs to damaged tissues; hence, they make the flow of ECM and the formation of neovascularization possible [96].

Among the two-dimensional and three-dimensional cell culture systems, three-dimensional systems are more effective than two-dimensional systems in mimicking the ECM in native tissues and provide a model for the regeneration of defects. They also improve adhesion and cell interactions, proliferation, the ECM production, repair of various tissues, and maintaining cell polarity to the system. Three-dimensional scaffolds can increase the sensitivity of stem cells to drugs and biomolecules, and optimizing their pore size promotes mechanical strength, thereby providing positive and dramatic results in tissue regeneration [183]. Three-dimensional nano-fibrous gelatin/silica bioactive glass hybrid scaffolds by creating a suitable microenvironment acts like a natural dental microenvironment and enhances the growth and differentiation of human DSCs [184]. Due to their flexible physical and mechanical properties and high biocompatibility, hydrogels are highly similar to the macromolecular components of the body as such they have been studied as an essential biomaterial [185, 186]. Hydrogels have a high potential to mimic ECM and are widely used due to their ability to provide gas and nutrient exchange in clinical settings [157, 187]. DSCs seem to improve tooth roots in combination with ECM scaffolding [188]. In immunocompromised mice, the transplantation of human DSCs with three-dimensional hydroxyapatite scaffolds containing peptide hydrogels induces internal vascular growth and osteodentin deposition, ultimately leading to the formation of the pulp tissue [189]. The nanofiber hydrogel PuraMatrix is used as a synthetic matrix to create a biocompatible, biodegradable, and non-toxic three-dimensional environment in a variety of cells [190]. DSCs injected with PuraMatrix into human root canals can differentiate into functional odontoblasts, which can heal damaged teeth through root formation [191]. Table 3 summarizes synthetic and ceramic scaffolds used in oral tissue engineering.

**Table 3:** Synthetic and ceramic scaffolds used in oral tissue engineering

No.	Author and Year	Defect location	Type of stem cell	Type of scaffold	Growth factor	Outcome
1	Schantz et al. [296]	Calvaria	BMSCs	PCL	–	After 3 months, the histology of the new islets showed new bone and blood vessels But the lesion did not heal completely
2	Petretta et al. [297]	In-vitro	BMSCs	PCL with Mg-doped bioactive glass	–	High level of biocompatibility, bioactivity, and cell adhesion have been observed
3	Wang et al. [298]	Dorsal subcutaneous space implantation	BMSCs	PCL	–	The osteogenesis of BMSCs was improved both in vitro and in vivo
4	Ren et al. [299]	Mandible	BMSCs	PLGA	–	After 3 months, histological studies showed complete repair of the bone lesion
5	Liu et al. [300]	Parietal	BMSCs	PLG	BMP2	New bone formation was observed after 12 weeks
6	Ma et al. [301]	In-vitro	BMSCs	PCL/PLGA/HA	–	The 3D printed scaffold showed good performance in mechanical and cell tests, suitable for bone tissue engineering
7	Pieri et al. [302]	Maxillary sinus	BMSCs	Flurohydroxyapatite	–	New bone formation was observed after 3 months
8	Kim et al. [303]	Mandible	BMSCs	HA/TCP	–	After 16 weeks, new bone formation around the implant was confirmed
9	Xu et al. [304]	Calvaria	-	TCP/PLGA	–	TCP/PLGA scaffold yielded more intact new bone for long-term repair of the defects
10	Zong et al. [305]	Calvaria	BMSCs	PLGA	–	After 20 weeks, histomorphometric examination showed new bone formation
11	Kim et al. [306]	In-vitro	BMSCs	PLGA/MH/ECM	Bioactive polydeoxyribonucleotide	Invreased osteogenesis, angiogenesis, adhesion, proliferation, and osteogenic differentiation of BMSCs
12	Zhao et al. [307]	Mandible	BMSCs	β-TCP	BMP 2	After 8 weeks, histometry showed new bone formation
13	Gao et al. [308]	Femor	BMSCs	β-TCP	–	Improved proliferation of BMSCs, glucose consumption and ALP activity
14	Nandi et al. [309]	Tibia	–	SiO2 and ZnO doped TCP	–	3D printing of TCP scaffolds improved bone formation. The addition of dopants in the TCP scaffolds improved osteogenic capabilities
15	Zhu et al. [310]	Mandibular condyle	BMSCs	PLGA	NELL-1	After 24 weeks mineral bone formation was indicated by μCT
16	Zou et al. [276]	Calvaria	BMSCs	Calcium Magnesium Phosphate Cement	HIF-1α	New bone formation was seen after 8 weeks
17	Thi Hiep et al. [311]	Bone defects	BMSCs	PCL/PLGA	BCP	new bone tissue replaced PCL/PLGA-BCP scaffold after 8 months of implantation

### Scaffold fabrication technologies

**Fig. 3:**
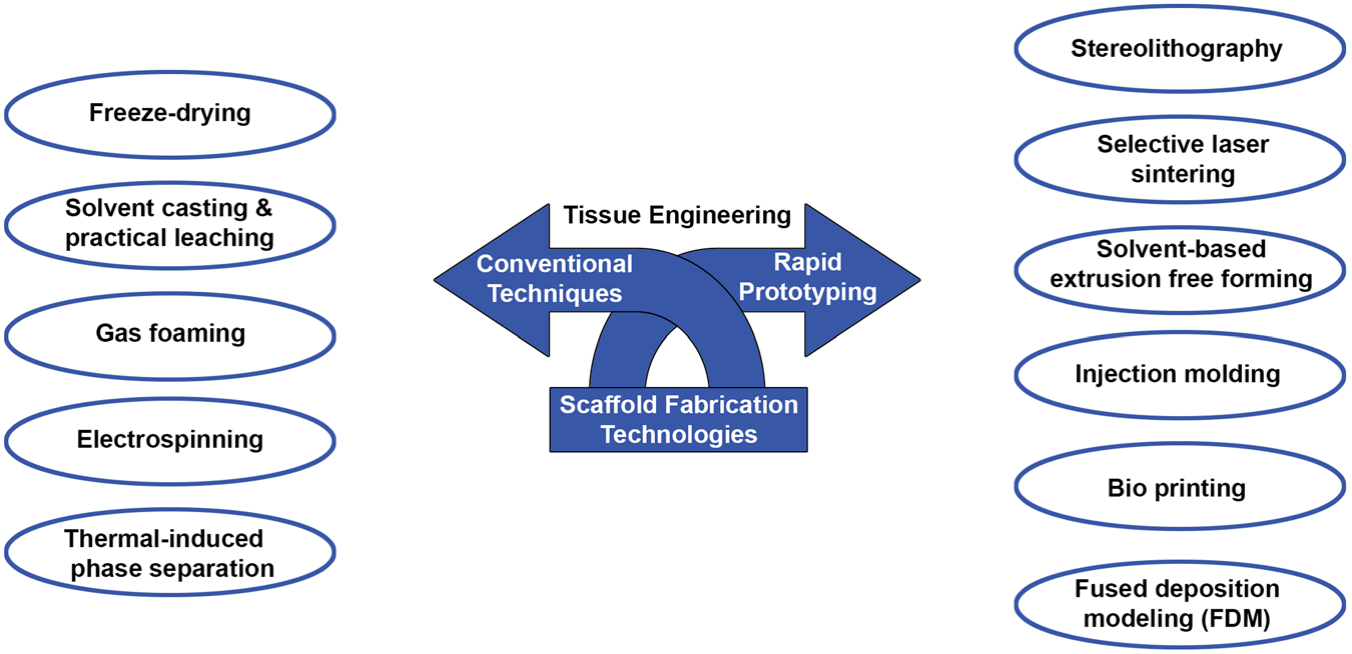
Classification of scaffold fabrication technologies in tissue engineering: conventional and rapid prototyping techniques

The techniques for the fabrication of 3D scaffolds are classified into conventional or rapid prototyping (RP) (Fig. 3).

#### Conventional techniques (Figure 4)

##### Solvent casting and particle leaching

In this technique, a solvent combined with uniformly-distributed salt particles of a certain size is used to dissolve the polymer solution. Solvent evaporates, leaving a matrix containing salt particles. The matrix is then submerged in water, and the salt leaches away to form a structure with high porosity [192, 193]. Solvent casting with particle leaching only suits thin membranes of thin wall three-dimensional specimens. Scaffolds developed by this method have a porosity of 50–90%. This technique is relatively easy and low-cost [194].

**Fig. 4:**
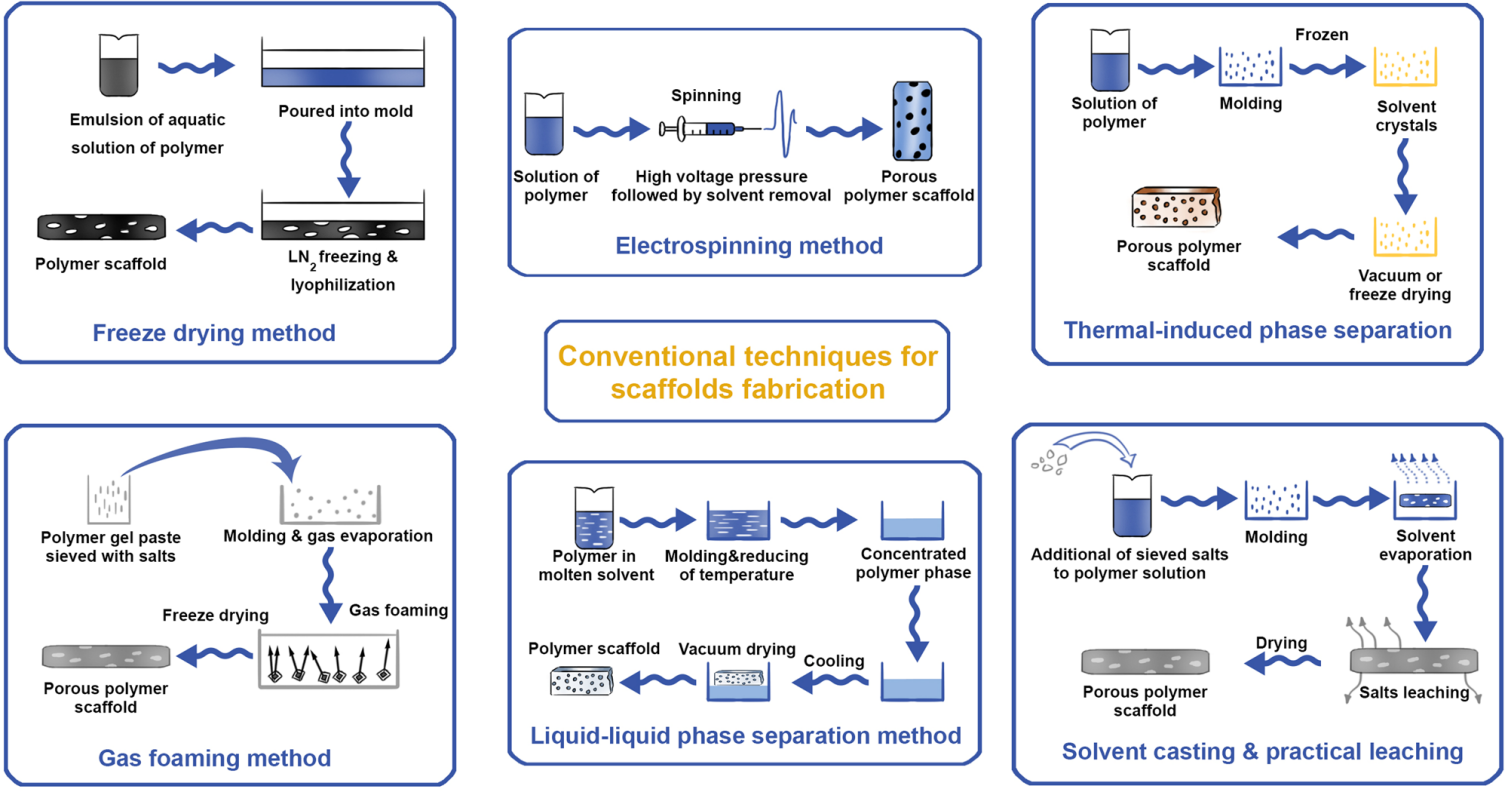
Schematic illustration of conventional techniques in scaffold fabrication

##### Freeze-drying

The freeze-drying process is also known as lyophilization and involves the use of a synthetic polymer, first dissolved in an appropriate solvent. After dissolution, the polymer solution is cooled under the freezing point, resulting in a solid solvent evaporated by sublimation to leave a solid scaffold with numerous interconnected pores [195]. In this technique, when the solution is cooled by the freezing point, the solutes can be separated in the ice phase, resulting in a small porous structure characterized by a “fence” of matter surrounding the ice. The scaffolds are achieved after consequent drying; and by simple dissolving and freeze-drying, the macro-porosity corresponds to the empty area initially occupied by ice crystals [196]. The benefit of this technique is the capability of obviating high temperatures, which could decrease the activity of integrated biological factors. Moreover, the pore size is managed by controlled and changing the freezing method

##### Stereolithography

The stereolithography method is basically used to create solid, three-dimensional objects by consecutively printing a thin layer of ultraviolet (UV) curable material layer-by-layer [197]. A stereolithography system has four main components: a tank with a photosensitive liquid resin, a transferable built platform, a UV laser for radiating resin, and a dynamic mirror system. The process begins with a UV laser by depositing a layer of photosensitive liquid resin on the platform. Following the solidification of the initial layer, the platform is lowered vertically. A second layer is then placed on the first layer; the process is repeated until a 3D scaffold is created. Finally, the uncured resin is cleaned off, and the scaffold is post-cured under UV light [198].

##### Gas foaming

The gas foaming technique is a technique to cope with using high temperature and organic cytotoxic solvents [199]. This technique uses relatively inert gas foaming agents such as carbon dioxide or nitrogen to pressurize modeled biologically degradable polymers with water or fluoroform until they are saturated or full of gas bubbles. This technique usually produces structures like a sponge with a pore size of 30–700 μm and a porosity up to 85% [200].

##### Electrospinning technique

The electrospinning technique offers ease and flexibility in controlling scaffold characteristics to suit various tissue engineering applications [201]. Moreover, electrospinning can deliver outstanding control of pore interconnectivity and internal and external scaffold geometry. In the basic principle of electrospinning, the polymer in a liquid phase is pumped via a thin needle of a specific diameter to assemble a conductive object, and when the required high voltage is realized and the applied electric power overpowers the surface tension forces of the used polymer solutions, a jet of polymer fibers is developed [202].

#### Rapid prototyping technology

Rapid prototyping (RP) technologies, also known as solid freeform fabrication, are widely applied in biomedical and tissue engineering applications. In this technique, the manufacturing method, with the aid of a specifically-designed computer-controlled 3D model, precise 3D scaffold models (based on Cad or CT scan files) are constructed by a layer-by-layer cyclic deposition and dispensation of material [203].

**Fig. 5:**
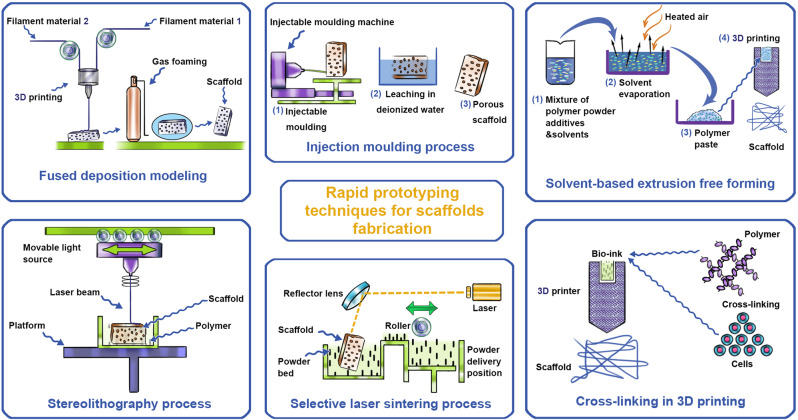
Schematic illustration of rapid prototyping techniques in scaffold fabrication

Various RP technologies in the market are as follows: three-dimensional printing (3DP), fused deposition modeling (FDM), stereolithography apparatus (SLA), and selective laser sintering (SLS) [204] (Fig. 5).

In the FDM technique, a solid polymer is cast into a hot extrusion nozzle to be melted and extruded on the surface of a 3D object using computer-controlled extrusion and deposition processes. The scaffold is made from multiple layers of adjacent microfilaments [205]. SLS was developed in 1986 by the Texas University of Austin. This technique uses the laser as the power source to sinter powdered material defined by a 3D model in thin layers. Due to using a laser, this technique has been utilized to make various materials such as polymers, metals, or ceramics [206].

##### Self-assembling technology

The current treatment practice mainly relies on inert biomaterials as substitutes for the decay of soft and mineralized tissues. However, lately, a tissue engineering method using a hydrogel scaffold seeded with two dental stem cell lines together with peptide-amphiphile (PA) was used to establish novel regenerative processes and regenerate dental tissues [207].

##### Three-dimensional printing (3DP)

Three-Dimensional Printing (3DP) is a process of creating tools, and functional prototype features directly from the computer models. It is a new fabrication method for tissue engineering, which can be utilized to control scaffold structure at the micron level precisely [208].

**Three-dimensional printing for regeneration of the tooth and tooth-supporting tissues**

Three-Dimensional bioprinting is a novel technology fundamentally derived from printing technology, which can print living cells directly into 3D structures [209]. The 3D printing technology is driving major innovations in regenerative dentistry [210]. The rise of 3D printing in dentistry has been parallel with CAD advancements and enhanced imaging techniques such as cone beam computed tomography (CBCT) and magnetic resonance imaging (MRI) to plan and print dental and maxillofacial prostheses to restore and replace lost structures [211]. The reconstruction of the complex system of the tooth and its supporting apparatus (like the ligament, alveolar bone, and cement) has been improved by 3D-printed bioengineered scaffolds [212]. 3D bioprinting boosts regenerative medicine and is being applied to address the need for tissues and organs suitable for transplantation. A wide range of biomaterials and printing strategies are used for 3D printing such as hydrogels, metals, ceramics, resins, and thermoplastics. Table 4 summarizes the material and techniques used in regenerative dentistry using the 3D printing technology.

**Table 4:** 3D printing technology, materials and techniques in regenerative dentistry

No.	Author and Year	Materials Used For 3D Printing	Technique of 3D Printing	Result
1	Chen et al. [312]	dECM-based bioink and PLGA Gelatin	Laser assisted bioprinting	dental pulp and tooth germ decellulised ECM demonstrated pleasant results in tooth regeneration
2	Osman et al. [313]	Zirconia	digital processing technique (DLP)	3D printed zirconia implants have good dimensional accuracy and mechanical properties similar to the conventionally produced ceramic implants
3	Tedesco et al. [314]	Titanium	DMLS	profitable bone growth and acceptable biocompatibility
4	Smith et al. [315]	Gelatin methacryloyl (GelMA)	Light-assisted printing	promising injectable hydrogel for the dental pulp and whole-tooth regeneration due to good biocompatibility and efficient revascularization
5	Ansari et al. [150]	Alginate hydrogels and MSCs	Extrusions technique	showed high cell viability, elasticity and porosity in alginate bioink as implant
6	Athirasala et al. [316]	Dentin-derived hydrogel.		favorable printability, cytocompatibility and natural odontogenic capacity
7	Lei et al. [317]	Platelet-Derived Growth factor (PRF)		osteoinductive and antibacterial factors, as well as Injectable PRF (I-PRF), accelerates the structure conforming to the defect
8	Yang et al. [318]	Ceramic		unique color and shape which optimized not only esthetics but also mechanics
9	Chang et al. [319]	Bio-Active^ITRI^	Laser-sintered 3D printing	active large bone formation on histomorphometric analysis
10	Mangano et al. [320]	Acrylic Resin & titanium	DMLS	Active prosthetic restoration for mandible
11	Park et al. [321]	Titanium	Digital processing technique (DLP)	3D printed implant was placed in a patient with an atrophic mandible due to osteoradionecrosis who received radiation treatment post squamous cell carcinoma resection
12	Höhne et al. [322]		Digital processing technique (DLP)	realistic differences in hardness, color, and different layers for enamel and dentin with a realistic pulp for education purposes in crown preparation
13	Barazanchi et al. [323]	Cobalt chromium alloy,	SLS	Compared to other metallic materials, CoCr alloy presents lower density, higher hardness and good corrosion resistance and bonding characteristics to porcelain

## Growth factors

Polypeptides that can stimulate cell proliferation and act as the major growth-regulatory molecules for cells in culture and in vivo are known as growth factors (GFs). Gfs, along with other morphogens comprise one of the three vital components in tissue engineering, which are combined with scaffolds and progenitor or stem cell population [213]. Various investigations have studied the use of recombinant growth factors separately or in combination with other growth factors or biomaterials for the regeneration of different oral tissues, including mandibular or maxillary bone [214], salivary glands [215], nerve regeneration [216], dentin–pulp complexes [217, 218], and periodontal tissued [219].

### Regenerative endodontics (Dentin–pulp complexes)

Adding signaling molecules and various growth factors to natural and artificial scaffolds can increase the regeneration of pulp-like tissues inside the canal by promoting dentin formation, mineralization, neovascularization, and innervation [220]. For example, DSCs linked to growth factor stromal cell-derived factor-1 (SDF-1) or granulocyte colony-stimulating factor (G-CSF) on a collagen scaffold, have promoted pulp regeneration in the animal pulpitis model [164, 221]. DSCs loaded on peptide hydrogels along with growth factors such as vascular endothelial growth factor (VEGF), TGF-β1, and FGF-1 can differentiate into odontoblast-like cells and vascularized dental pulp-like tissue within the dentin cylinder [222]. DSCs isolated from adult human dental pulp implanted on the surfaces of three-dimensional collagen gel cylinders show significant cellular uptake when combined with BMP-7, SDF-1α, and bFGF [223]. Furthermore, SDF, FGF, TGF-β1, VEGF, and BMP as growth factors, when loaded on scaffolds such as peptide hydrogels, collagen, gelatin hydrogels, and alginate hydrogels, enhance the endodontic regeneration of DSCs [224]. The combination of SDF-1 with biomaterials to use different endogenous stem cells is highly effective. A study revealed SDF-1 embedded in a silk fibroin scaffold resulted in pulp regeneration through DPSC induction in a pulpectomized mature canine preclinical model [225]. Further, SDF-1 induces and regenerates the structure of pulpdentin by absorbing and transferring SCAP from the apex to the root canal space [226]. The implantation of DSCs with poly-ε-caprolactone and hydroxyapatite along with SDF-1 and BMP-7 results in tooth-like structures in the mandibular incisor extraction socket [227]. The two growth factors, G-CSF and FGF-2, have the greatest impact on the migration of SCAPs. previous studies have revealed that combining G-CSF with TGF-β1 leads to the migration and high biomineralization of endogenous SCAPs in root canal repair methods. G-CSF also has stimulatory effects on the movement of DPSCs from adult teeth. These mobilized DPSCs have higher vascularity and pulp regeneration ability than colony-derived DPSCs [228]. DSCs implanted in a collagen/chitosan scaffold containing a non-cellular ECM result in the expression of dentin sialoprotein in nude mice, which ultimately produce the pulp-like tissue in the tooth [229]. Observations have indicated that the co-culture of DSCs with other stem cells improves neovascularization, and the co-culture of DSCs and human umbilical vein endothelial cells with gelatin methacrylate xenogeneic hydrogel leads to the formation of new vascular pulp in rat teeth [230]. Dissanayaka et al. found that the transplantation of DSCs and human umbilical vein endothelial cells into PuraMatrix containing VEGF increased the vascularization and mineralization of mouse vascularized pulp-like tissue and osteodentin [231]. Woloszyk et al. also reported that the use of silk fibroin scaffolds increased the ability of human DSCs to attract vessels, thereby improving and regenerating damaged tissues [232]. Yang et al.‘s study showed that the transplantation of DSCs with a piece of silk fibrin tooth/scaffold loaded with SDF-1 resulted in the formation of pulplike tissues with vascularity, the formation of an organized fibrous matrix, and the formation of dentin in the nude mice [225].

### Periodontal and alveolar bone regeneration

Many studies have addressed bone regeneration [233, 234], according to which the ossification capacity of DSCs varies depending on their origin (i.e., dental pulp, tooth follicle, gingival tissue, and periodontal ligament), which can change the ossification ability of DSCs depending on the selected biomaterial scaffolds [235, 236]. For example, the ability to repair bone defects is greater in the DSCs of periodontal ligament origin encapsulated in an arginine glycine-aspartic acid tripeptide scaffold [237]. DSCs derived from dental pulp have a high potential for neovascularization, and, due to their ability to differentiate into osteoblasts, they can enhance bone repair [238]. One of the most common known scaffolds in bone tissue engineering to seed DSCs with human dental pulp or exfoliated deciduous teeth origin is included collagen sponge membranes (to repair defects in the human mandible bone) and hydroxyapatite/tri-calcium phosphate ceramic granules [14]. Hernández-Monjaraz et al.‘s study showed that in patients with periodontal problems, DSCs implanted on collagen-polyvinylpyrrolidone sponge scaffold increased bone density and decreased tooth mobility and periodontal pocket depth in the bone defect area [239]. Tanikawa also managed to reconstruct bone and fill alveolar defects in cleft lip and palate patients through DSCs with a hydroxyapatite-collagen sponge scaffold [240]. In a study, Chamieh et al. found that DSCs implanted in dense collagen gel scaffolds had a greater effect on the healing process of the skull and face than cell-less scaffolds [241]. Ferrarotti et al. used DSCs implanted in collagen sponges to treat patients with chronic periodontitis with deep intraosseous defects, which significantly improved periodontal regeneration [242]. The important point in a successful cell transplant is the optimal number of DSCs. Moreover, the composition of the scaffold and its surface properties play a critical role in the bone differentiation of DSCs and the process of bone tissue regeneration [238, 243]. For example, DSCs implanted in a type I collagen matrix, fibrin, hyaluronic acid, and polyesteramide type-C play a vital role in mineralization [244]. Due to ceramic scaffolds’ chemical and structural similarity to native bone, they are commonly used to enhance bone regeneration and repair DSCs [245]. Strong bone formation in the femoral bone defect area of rats was observed after applying DSCs implanted in bioactive glass nanoparticles/chitosan-gelatin bionocomposite compared to mesoporous bioactive glass nanospheres [246]. Some biomaterial scaffolds facilitate biomolecule-induced tissue formation. Fu showed that the 3D matrix scaffold enriched with DSCs in nude mice increased BMP-9-induced osteogenesis and mineralization in ectopic bones [247].

### Nerve regeneration

In addition to the abovementioned points, DSCs can differentiate into neuron-like cells, Schwann, glia, and oligodendrocytes [248]. Various studies have indicated that the implantation of DCSs in different scaffolds increases the lifespan of cells and their differentiation into neuronal-like cells [249, 250]. The use of combined DSCs with different scaffolds, including chitosan, heparinpoloxamer, silicone tubes, and poly-ε-caprolactone/ poly-lactide-co-glycolic acid, improves the function of damaged nerve tissues and reduces inflammatory responses [251]. For example, in experimental models of spinal cord injury, the transplantation of DSCs with chitosan scaffolds enhanced motor function and suppressed inflammatory responses, in which glial cell-derived neurotrophic factors and brain-derived neurotrophic factors seem to play a vital role. Combining DSCs with scaffolds also reduces caspase activity, thereby preventing cell damage and death [252]. Human DSCs isolated from periodontal ligament gingival tissues and enclosed in three-dimensional alginate and hyaluronic acid scaffolds in the presence of nerve growth factor (NGF) differentiate DSCs to neural tissues [253]. Human DSCs with the expression strength of STRO-1, c-Kit, and CD34 markers, when implanted on collagen scaffolds, could have axonal regeneration from proximal to distal stumps in mice with sciatic nerve defects [63].

**Fig. 6:**
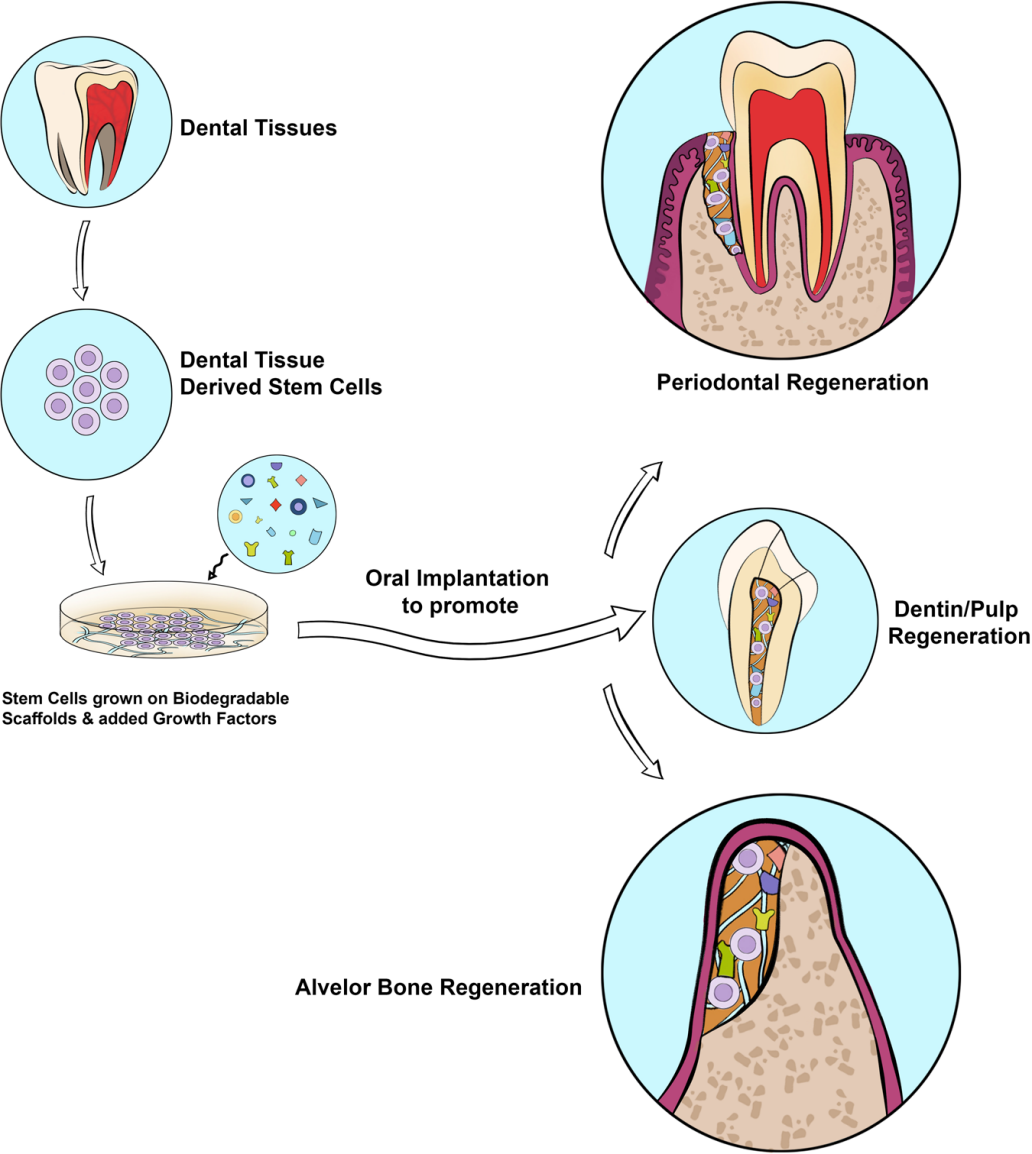
Application of dental tissue-derived stem cells combined with growth factors and scaffolds in dentistry

Figure 6 demonstrates the application of dental tissue-derived stem cells combined with growth factors and scaffolds in oral regenerations.

## Discussion

In recent years, there have been many studies on stem cell therapy. This field is revitalizing in various fields of medicine such as dentistry and medical diseases. Because the oral and maxillofacial areas are a promising source of SCs, physicians and dentists need to have adequate and up-to-date information on SCs recognition and access during patients’ treatment. According to such studies, different types of DSCs have been introduced [101, 254], all of which are well suited for early research in resuscitation medicine. Preclinical studies and some clinical trials have yielded successful results regarding the use of DSCs. It has been observed that tooth SCs are safe and supportive for regenerating lost or damaged tooth tissues [255–257].

Most SCs used in dentistry come from dental structures such as dental/apical papilla, PDLs, and even decayed deciduous teeth. These dental cells have features such as a high proliferation rate, wide differentiation potential in different mesenchymal cell lineages, and weak immunogenic effects, making them special in regenerative medicine and dentistry [254].

The results of various studies show the strong potential of DSCs in the production of dental components such as dentin, pulp, cement, and periodontal ligament associated with the presence of odontoblasts and cementoblasts. For example, some DSCs can form chondrocytes, osteocytes, neurons, and adipocytes in vitro. According to the research findings, DSCs such as DPSCs can regenerate dentin/pulp [221, 258, 259], SHEDs and DFPCs can strengthen bone [83, 256, 260], and PDLSCs play a role in periodontal regeneration [101, 257, 261].

Before using DSCs for tissue regeneration, the key point is to find reliable ways to control previous inflammatory environments. Further studies are needed to elucidate the underlying mechanisms of lost tissue regeneration and the immune system modifying features of the DSCs, followed by human clinical trials [78].

## Conclusion

Recently, stem cell-mediated therapeutic interventions have received much attention and have made significant advancement in treating diseases, especially those not cured by conventional methods. Although many studies have addressed the use of biomolecules with appropriate scaffolds to treat effective cell transplantation with DSCs and have yielded significant results, there is still a long way to identify these molecules for better therapeutic outcomes and their interaction with ECMs and DSCs. Importantly, the focus is on the innovative combinations of biomaterials and biomolecules to enhance the ability of DSCs to provide new therapeutic approaches. Stem cell transplantation is a promising option; however, at the moment, it cannot be considered a therapeutic miracle. In general, although the SCs of dental origin have many applications they also have certain limitations. One of its main limitations is the difficulty in identifying, isolating, purifying, and growing these cells continuously in laboratories. Rejection by the immune system is another problem requiring further thorough investigation. However, autologous cells can help solve this problem. SCs research in dentistry has its own challenges and risks, and this necessitates further research.

## Abbreviations

Stem cells: (SCs)
Dental stem cells: (DSCs)
Pluripotent Stem Cells: (PSCs)
Induced Pluripotent Stem Cells: (iPSCs)
Embryonic Stem Cells: (ESCs)
Bone Marrow-Derived MSCs: (BMSCs)
Oral epithelial stem cells: (OESCs)
Mesenchymal stem cell: (MSCs)
Dental Pulp SCs: (DPSCs)
Dental Follicle Progenitor Cells: (DFPCs)
Stem cells from Exfoliated Deciduous Teeth: (SHED)
Stem cells from Apical Papilla: (SCAP)
Tooth Germ stem cells: (TGSCs)
Periodontal Ligament stem cells: (PDLSCs)
Tooth germ progenitor cells: (TGPCs)
Gingival mesenchymal stem/progenitor cells: (GMSCs)
Human leukocyte antigen G: (HLA-G)
Hepatocyte growth factor: (HGF)
Transforming growth factor beta: (TGF-β)
Interleukin-6: (IL-6)
Prostaglandin E2: (PGE2)
Indole amine 2, 3-dioxygenase: (IDO)
Nitric oxide: (NO)
Bone morphogenic protein 2: (BMP-2)
Bone morphogenic protein 7: (BMP-7)
Oral mucosa stem cells: (OMSCs)
Adipose Tissue-Derived Stem Cells: (ASCs)
Extra cellular matrix: (ECM)
Stromal cell derived factor-1: (SDF-1)
Granulocyte colony-stimulating factor: (G-CSF)
Vascular endothelial growth factor: (VEGF)
Fibroblast growth factor: (FGF)
Nerve growth factor: (NGF)
Cone beam computed tomography: (CBCT)
Magnetic resonance imaging: (MRI)
Rapid prototyping: (RP)
Ultraviolet: (UV)
Three-dimensional printing: (3DP)
Fused deposition modeling: (FDM)
Stereolithography apparatus: (SLA)
Selective laser sintering: (SLS)
Peptide-amphiphile: (PA)
Growth factors: (GFs)
